# Evolutionary Histories of *Camellia japonica* and *Camellia rusticana*

**DOI:** 10.1002/ece3.70721

**Published:** 2024-12-24

**Authors:** Harue Abe, Saneyoshi Ueno, Ayumi Matsuo, Shun K. Hirota, Hiroki Miura, Mong‐Huai Su, Yun‐Guang Shen, Yoshihiko Tsumura, Yoshihisa Suyama, Zhong‐Lang Wang

**Affiliations:** ^1^ Center for Sustainable Agriculture and Forestry, Faculty of Agriculture Niigata University Sado Niigata Japan; ^2^ Department of Forest Molecular Genetics and Biotechnology, Forestry and Forest Products Research Institute Forest Research and Management Organization Tsukuba Ibaraki Japan; ^3^ Field Science Center, Graduate School of Agricultural Science Tohoku University Osaki Miyagi Japan; ^4^ Botanical Gardens Osaka Metropolitan University Katano City Osaka Japan; ^5^ Aomori Prefectural Asamushi Aquarium Aomori Aomori Japan; ^6^ Department of Forestry and Nature Conservation Chinese Culture University Taipei Taiwan; ^7^ Kunming Botanical Garden, Kunming Institute of Botany, Chinese Academy of Sciences Kunming China; ^8^ Faculty of Life and Environmental Sciences University of Tsukuba Tsukuba Ibaraki Japan; ^9^ Graduate School of Agricultural Science Tohoku University Osaki Miyagi Japan

**Keywords:** *Camellia japonica*, *Camellia rusticana*, evolution, genetic diversity, phylogeny, Theaceae

## Abstract

The genus *Camellia* is widely distributed, primarily in East Asia. *Camellia japonica* is located at the northern limit of this genus distribution, and understanding changes in its distribution is crucial for understanding the evolution of plants in this region, as well as their relationship with geological history and climate change. Moreover, the classification of sect. *Camellia* in Japan has not been clarified. Therefore, this study aims to understand the evolutionary history of the Japanese sect. *Camellia*. The genetic population structure was analysed using SNP data and MIG‐seq. The relationship between the Japanese sect. *Camellia*, including the related species in China, was further inferred from the phylogeny generated by RA x ML, SplitsTree and PCA. Population genetic structure was inferred using a Bayesian clustering method (ADMIXTURE). We subsequently employed approximate Bayesian computation, which was further supported by the coalescent simulations (DIYABC, fastsimcoal and Bayesian Skyline Plots) to explore the changes in population, determining which events appropriately explain the phylogeographical signature. Ecological niche modelling was combined with genetic analyses to compare current and past distributions. The analyses consistently showed that *C. japonica* and *C. rusticana* are distinct, having diverged from each other during the Middle to Late Miocene period. Furthermore, *C. japonica* differentiated into four major populations (North, South, Ryukyu‐Taiwan and Continent). The Japanese sect. *Camellia* underwent speciation during archipelago formation, reflecting its ancient evolutionary history compared with other native Japanese plants. *C. rusticana* did not diverge from *C. japonica* in snow‐rich environments during the Quaternary period. Our results suggest that both species have been independent since ancient times and that ancestral populations of *C. japonica* have persisted in northern regions. Furthermore, the *C. japonica* population on the continent is hypothesised to have experienced a reverse‐colonisation event from southern Japan during the late Pleistocene glaciation.

## Graphical abstract

The Japanese sect. *Camellia* underwent speciation during archipelago formation, reflecting its ancient evolutionary history compared with other native Japanese plants. *Camellia rusticana* did not diverge from *C. japonica* in snow‐rich environments during the Quaternary period. Our results suggest that both species have been independent since ancient times and that ancestral populations of *C. japonica* have persisted in northern regions. Furthermore, the *C. japonica* population on the continent is hypothesised to have experienced a reverse‐colonisation event from southern Japan during the late Pleistocene glaciation.

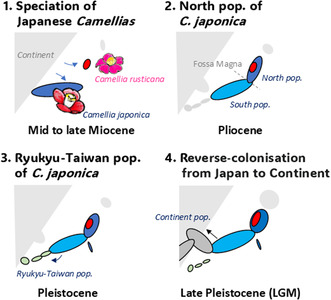

## 1 Introduction

Theaceae family comprises approximately 28 genera and 600 species, predominantly distributed in humid temperate regions (Nagamasu 2006). *Camellia* is a pivotal genus identified in the Sino‐Japanese Floristic Region (SJFR) of East Asia, with approximately 120 species distributed in East and Southeast Asia. The Sino‐Japanese floristic region (SJFR) is renowned for its high levels of biodiversity and endemism, primarily attributed to its complex climatic history and varied topography. This region has served as a refuge during past climatic fluctuations, facilitating the survival and diversification of many plant species (e.g., Lu et al. 2020; Qiu, Fu, and Comes 2011; Takahashi et al. 1994). The historical biogeography and climate change relationships of *Camellia* have been investigated (e.g., Zan et al. 2023); however, the evolutionary history of sect. *Camellia* in Japan remains unexplored. Investigations for the molecular phylogeny of sect. *Camellia* species in their northernmost limit of distribution in Japan are warranted to comprehend the evolution, climate change association and prospective conservation of temperate forest tree species.

The *Camellia* section *Camellia* characteristically features strikingly aesthetic large red petals adapted for bird pollination. The seeds are primarily dispersed by gravity, with secondary dispersal by animals, particularly rodents (Abe et al. 2006). The Japanese species *Camellia japonica* possesses multiple horticultural cultivars. The divergence of *C. japonica* from *C. chekiangoleosa* is estimated to have occurred within a timeline ranging from approximately 10 to 17 Ma (Cheng et al. 2022; Yan et al. 2021; Rao et al. 2018). This implies that *C. japonica* differentiated from its Eurasian relatives during the Japanese archipelago formation. Elucidating the demographic history of this northernmost *Camellia* species in temperate forests is essential for predicting plant dispersal in response to geographical events and climate change in Japan. For example, the genetic differentiation of organisms, divided by the Fossa Magna on Honshu, highlights the geological and biological distribution alterations in the Japanese archipelago. Quaternary climatic fluctuation studies have provided phylogeographic insights into glacial refugia, colonisation routes and range expansion (Dumolin‐Lapègue et al. 1997; Gonzales, Hamrick, and Chang 2008; Ikeda and Setoguchi 2007; McLachlan et al. 2007; Petit et al. 2003). Nonetheless, the quaternary expansion–contraction model remains controversial, with recent research supporting ‘cryptic (or micro‐) refugia’ at higher latitudes (Provan and Bennett 2008; Rull 2009). Quaternary climate shifts and the associated environmental modifications have facilitated range fragmentation, vicariance and population isolation, thereby fostering allopatric (incipient) speciation through selection and genetic drift (Comes, Tribsch, and Bittkau 2008; Yesson, Toomey, and Culham 2009).

*Camellia japonica* and *C. rusticana* from sect. *Camellia* exhibits distinct natural distributions in Japan (Figure S1). *C. japonica* is predominantly localised in the warm coastal regions of the Ryukyu Islands, Kyushu, Shikoku and Honshu (Horikawa 1972) and Taiwan, South Korea and the coastal areas of mainland China (Nagamasu 2006). Conversely, *C. rusticana* occurs in the snow‐clad regions by the Sea of Japan side, from Shiga to Akita Prefecture, earning it the nickname ‘snow *Camellia*’. Owing to variations in habitats and morphologies (e.g., Ishizawa 1988; Tsuyama 1956), these species are disparately categorised by researchers, causing taxonomic uncertainty stemming from known hybridisation in adjacent habitats (Ueno, 2009). Tsuyama (1956, 1988) suggested that the ‘snow *Camellia*’ presumably adapted and underwent speciation from a temperate climate to frigid environments, as closely related species are distributed in the warm temperate zone along the Pacific Ocean (Sakai 1982). Two varieties of *Daphniphyllum macropodum* genetically support the aforementioned adaptation (Yoichi et al. 2023). However, conflicting findings have emerged from prior studies comparing the molecular phylogenies of *C. japonica* and *C. rusticana*. PCR‐restriction fragment length polymorphism analyses revealed standard bands between *C. chekiangoleosa* and *C. rusticana*, whereas *C. japonica* exhibited disparate patterns (Tanikawa et al. 2008). Vijayan, Zhang, and Tsou (2009) positioned *C. chekiangoleosa* as holding an ancestral position within the same clade relative to *C. japonica* and *C. rusticana*, which are identified as closely related species from the continent. Zhao, Hodkinson, and Parnell (2022) did not include *C. rusticana*; nonetheless, *C. chekiangoleosa* was positioned as a sister species to *C. japonica* in their broader phylogenetic analysis of the *Camellia* section. Molecular phylogenetic studies on *Camellia* depicted speciation within the sect. *Camellia* predates the glacial period, with its origin and diversification occurring 6–30 million years ago (Ma) during the Miocene epoch (Cheng et al. 2022; Qin et al. 2023; Rao et al. 2018; Wu et al. 2022; Zan et al. 2023; Zhang et al. 2014, 2022; Zhao, Hodkinson, and Parnell 2022). These uncertainties warrant subsequent analyses involving ancestral Eurasian species, which are crucial for delineating the migration time of the sect. *Camellia* in Japan and their speciation timing and processes in a geohistorical context (Abe, Miura, and Katayama 2023); nevertheless, their precise evolutionary relationships remain ambiguous.

Based on the above background, this study aims to analyse the demographic history of *C. japonica* and *C. rusticana* and clarify the impact of geological history and climate change on their distribution and evolution. This will highlight the importance of preserving the genetic diversity of the sect. *Camellia* in Japan.

## 2 Methods

### 2.1 Materials

Leaves were collected for DNA extraction from 91 populations of *C. japonica* and *C. rusticana*, including hybrids. Species identification was contingent upon the morphology of flowers and leaves, using the methodology outlined by Abe, Miura, and Motonaga (2021). We included *C. azalea*, *C. edithae*, *C. oleifera* and *C. fluviatilis* in the following analysis, which are potentially closely related to the Japanese *Camellia* section (Li et al. 2021; Rao et al. 2018; Vijayan, Zhang, and Tsou 2009), and treated *Camellia chekiangoleosa* as the closest continental species. *C. chekiangoleosa* was acquired from six individuals from two distinct populations, along with samples from botanical gardens. The distance between the aforementioned individuals was maintained at ≥ 20 m to avoid collection from clonal individuals. *Camellia impressinervis* and *C. limonia* (both sect. Chrysantha) were obtained from a botanical garden for outgroup species (Table S1). An average of 3.7 individuals (range 1–10) from all *C. japonica* and *C. rusticana* populations (total 349 individuals), including *C. chekiangoleosa* and two outgroup species, were genetically analysed for genome‐wide single‐nucleotide polymorphism (SNP) detection. The sample locations and numbers and analytical methods are listed in Table S1.

DNA was extracted from the leaves using the CTAB method (Murray and Thompson 2008).

### 2.2 Genome‐Wide SNP Analysis

SNPs were identified using multiplexed inter‐simple sequence repeat (ISSR) genotyping by sequencing (MIG‐seq) (Suyama and Matsuki 2015; Suyama et al. 2022). An MIG‐seq library was prepared and sequenced with the established protocol (Suyama et al. 2022). ISSRs were amplified using primer set‐1 (Suyama and Matsuki 2015), and the index and adaptor sequences were incorporated at both ends of the PCR products in the first and second PCR, respectively. The Illumina MiSeq platform and MiSeq Reagent Kit v3 (150 cycles; Illumina, San Diego, CA, USA) were used to sequence 80 base paired‐end reads.

Low‐quality and adapter sequence‐bearing extremely short reads were removed using Trimmomatic 0.39 (Bolger, Lohse, and Usadel 2014). The Stacks v2.52 pipeline (Catchen et al. 2011; Rochette, Rivera‐Colón, and Catchen 2019) was used to obtain individual genotypes. The GSTACKS program in Stacks employing the *C. sinensis* CSS_ChrLev_20200506 genome reference sequence at Tea Plant Information Archive (TPIA) was used for genotyping. The following option settings were used with the populations command: the minimum percentage of individuals across populations (*R*) = 0.5, the maximum observed heterozygosity, ‐‐max‐obshet = 0.6; minimum minor allele frequency, ‐‐minmaf = 2/the number of individuals. *R* values of 0.1, 0.3, 0.5 and 0.8 were tested, and the best value was selected based on bootstrap support from maximum likelihood (ML) phylogenetic trees. We also performed de novo assembly under the same settings; however, it resulted in significantly fewer shared loci compared to mapping with the reference genome (Table S3). Additionally, we used the mapped data in this study because accurate values for mutation rates and other estimates were available for *C. sinensis* .

### 2.3 Phylogenetic Analyses

The populations command in Stacks was employed to estimate pairwise *F*_st_ values for each clade and calculate *H*_*e*_ indices based on fixed and variant sites. The concatenated SNP matrix was subjected to phylogenetic analysis using the RA x ML version 8.2.11 (Stamatakis 2014) with the GTRGAMMA model and visualised using FigTree v. 1.4.3 (Rambaut 2014). The NeighborNet network algorithm was administered using the SplitsTree4 software (Huson and Bryant 2006) to obtain an SNP distance matrix. Subsequently, for principal component analysis (PCA), the VCF file was filtered using PLINK 1.90 (Purcell et al. 2007) in linkage disequilibrium with the following parameters: make‐bed‐indep‐pairwise 50 10 0.1—allow‐extra‐chr.

### 2.4 Genetic Structure Determination

The population structure of each species was determined using ADMIXTURE version 1.3.0 (Alexander and Lange 2011). The optimal post‐simulation *K* values (MedMedK, MedMeanK, MaxMedK and MaxMeanK) were estimated using the Puechmaille method‐based (Puechmaille 2016) online software Structure Selector (Li and Liu 2018), which outperformed the conventional Evanno, Regnaut, and Goudet (2005) methodology for uneven population sample sizes.

### 2.5 Population Demographic History

The DIYABC Random Forest v1.0 (Cornuet et al. 2014), an approximate Bayesian computation (ABC) method assessing historical changes in population size, inferred the demographic histories of the three species and specific populations of *C. japonica* . The ADMIXTURE analytical results were corroborated by a demographic analysis using samples that excluded inter‐specific hybridisation in the three species and inter‐population hybridisation of *C. japonica* . The demography of the three species was comprehended by estimating the optimal models for all probable scenario combinations (10 scenarios; Figure S2) without assuming any hybridisation. Furthermore, among the top three scenarios, model selection was repeated. A comparable model selection was performed for *C. japonica* , employing the northern ancestral population. Six scenarios (Figure S3) were selected for the inter‐population demographic analysis of *C. japonica* using the four populations estimated from the ADMIXTURE analysis. The lifespan (generation time) of *C. japonica* was assumed to be ≥ 10 years (Kubayashi et al. 2010). To generate a posterior distribution, 20,000 simulations were performed per scenario, setting N# to 10–100,000 individuals and Time# to 10–2,000,000 or 500,000 generations (Table S2). The maximum generation time in the molecular phylogenetic analysis by Zhao, Hodkinson, and Parnell (2022), using four fossil calibrations for molecular dating, was attributable to the divergence age between *C. japonica* and its sister species, *C. chekiangoleosa*, estimated at 9.9 Ma, which necessitated an older age. All parameters were uniform before distribution. For scenario selection, 10,000 and 1000 trees were used to establish a random forest classification and estimate parameters, respectively. The compatibility between scenarios, associated priors and observed data was assessed using linear discriminant analysis (LDA).

Recognising that DIYABC‐RF accurately determines scaled parameters such as t/Ne yet fails to estimate absolute parameter values due to the lack of molecular evolutionary rates, we conducted further analysis using fastsimcoal2.7 (Excoffier et al. 2021) on the scenarios refined by DIYABC‐RF. During this analysis, we incorporated gene flow between *C. japonica* and *C. rusticana* into the model, and for the divergence model between *C. japonica* populations, we also incorporated gene flow between the North and South pops of Honshu. Furthermore, we estimated the divergence times in a simple branching model for *C. japonica* and *C. rusticana*, excluding *C. chekiangoleosa*. Each parameter is shown in Table S5. For fastsimcoal2.7 analyses, we calculated two‐ and one‐dimensional folded site‐frequency spectra (SFS). The SFS was estimated using easySFS (https://github.com/isaacovercast/easySFS). To maximise the number of SNPs available for SFS estimation, we employed a down‐projection method. The unfolded multidimensional site‐frequency spectrum (multiSFS) was generated using easySFS, following the recommended down‐projection approach to maximise the number of segregating sites while accounting for missing data (Rosser et al. 2024). We used the values estimated from the reference genome of *C. sinensis* , with a generation time of 3 years and a mutation rate per generation per site of 6.5 × 10^−9^ (Wei et al. 2018; Zhang et al. 2021). The genome size of *C. sinensis* ranges from 3.08 Gb to 3.2 Gb (Wang et al. 2022; Xia et al. 2017), while *C. japonica* and *C. chekiangoleosa* exhibited smaller genomes of approximately 2.3 Gb (Huang et al. 2013). The generation time for *C. japonica* is estimated to be over 10 years (Kubayashi et al. 2010). The actual evolutionary rate is likely to be slower than the rate estimated for *C. sinensis* because mutation rates tend to increase with genome size (Lynch 2010). Therefore, we consider divergence times adjusted for the generation time exceeding 10 years in *C. japonica* . For each of the scenarios, we initially conducted 50 independent iterations with 200,000 coalescent simulations and 60 optimisation cycles to obtain point estimates of the demographic parameters based on the highest maximum composite likelihood. The overall best estimate parameters set was used to generate 50 pseudo‐observed SFSs (for a similar number of polymorphic SNPs) for non‐parametric bootstraps with the same simulation settings. The estimated parameter values from the top 50 runs were used to calculate 95% confidence intervals.

The demographic population history was reconstructed using the coalescent Bayesian skyline plot (BSP) in BEAST 2.7.7 (Bouckaert et al. 2014, 2019), which uses MCMC to infer rooted, time‐measured phylogenetic trees. For the substitution rate estimation, we used the mutation rate of *C. sinensis* as a strict clock rate. A standard deviation was applied in a normal distribution using BEAUTi as part of the BEAST package. The chain length was initially set to 10,000,000 MCMC iterations, and if convergence was not achieved, it was extended to 30,000,000 MCMC iterations with sampling every 10,000 generations. We assessed runs using Tracer 1.7.2 (Rambaut et al. 2018) to examine convergence (Effective Sample Size > 1000) and tree topologies. In the comparison between *C. japonica* and *C. rusticana*, the North pops and South pops of *C. japonica* from Honshu were used.

### 2.6 Distribution Shift Reconstruction

The historical distribution transition between *C. japonica* and *C. rusticana* from the past to the present was compared by estimating suitable habitats based on the last glacial maximum (LGM) of 22,000 years and current environmental conditions using MaxEnt, an analytical software for estimating ecological niche models (ENMs; Phillips, Anderson, and Schapire 2006). The analysis was performed using species distribution information from Japan, as detailed in Table S1. The selection of environmental variables was based on Sakaguchi et al. (2012), who used various combinations, including annual average temperature, average temperature during the four summer months, average temperature during the four winter months, annual precipitation, precipitation during the four rainy months and precipitation during the four dry months. Additionally, considering the importance of winter precipitation for *Camellia*, different combinations were explored and the combination that best matched the current distribution was utilised. The environmental variables were estimated from the Community Climate System Model 3.0 (Collins et al. 2006) for the final glacial period using a 4.5‐km mesh size in WorldClim v. 1.4 (Hijmans et al. 2005). The resulting distribution prediction model was computed from the area under the curve (AUC) using receiver operating characteristic (ROC) curve analysis (Fawcett 2006). Randomly sampled background points in the study area represented environmental conditions in areas without species presence, serving as pseudo‐absence data in MaxEnt. Model performance was assessed using ROC analysis, with AUC values ranging from 0 to 1; values ≈1 indicated higher accuracy. All layers, including environmental variables, were prepared with a uniform cell size of 1 km^2^ using ArcGIS software.

## 3 Results

### 3.1 Genome‐Wide SNPs

The phylogenetic relationships and population structures of the three *Camellia* species and *C. japonica* varieties were compared using MIG‐seq with a high‐throughput sequencer. The nuclear genome timescale is considerably distinct from the chloroplast genome timescale, with the nuclear genome evolutionary processes being more rapid (Drouin, Daoud, and Xia 2008). Following reference genome‐based genotyping, a total of 92,724, 41,665, 20,462 and 3800 SNPs in all samples with *R* = 0.1, 0.3, 0.5 and 0.8, respectively, and 13,449 in *C. japonica* with *R* = 0.5, were obtained.

### 3.2 Phylogeny

The molecular phylogenetic analytical results of all 349 samples were categorised into six taxonomic entities: outgroup, *C. chekiangoleosa*, *C. rusticana*, the Taiwan regions, the Ryukyu regions and the other areas of *C. japonica* (Figure S4). *C. chekiangoleosa*, *C. rusticana* and *C. japonica* were separated with 100% bootstrap value (BS) support. Three distinct groups pertaining to the clade of *C. japonica* were separated, distinguishing the Taiwanese population with a BS of 91 and the Ryukyu Island population with a BS of 57 from the rest.

### 3.3 Admixture

**FIGURE 1:**
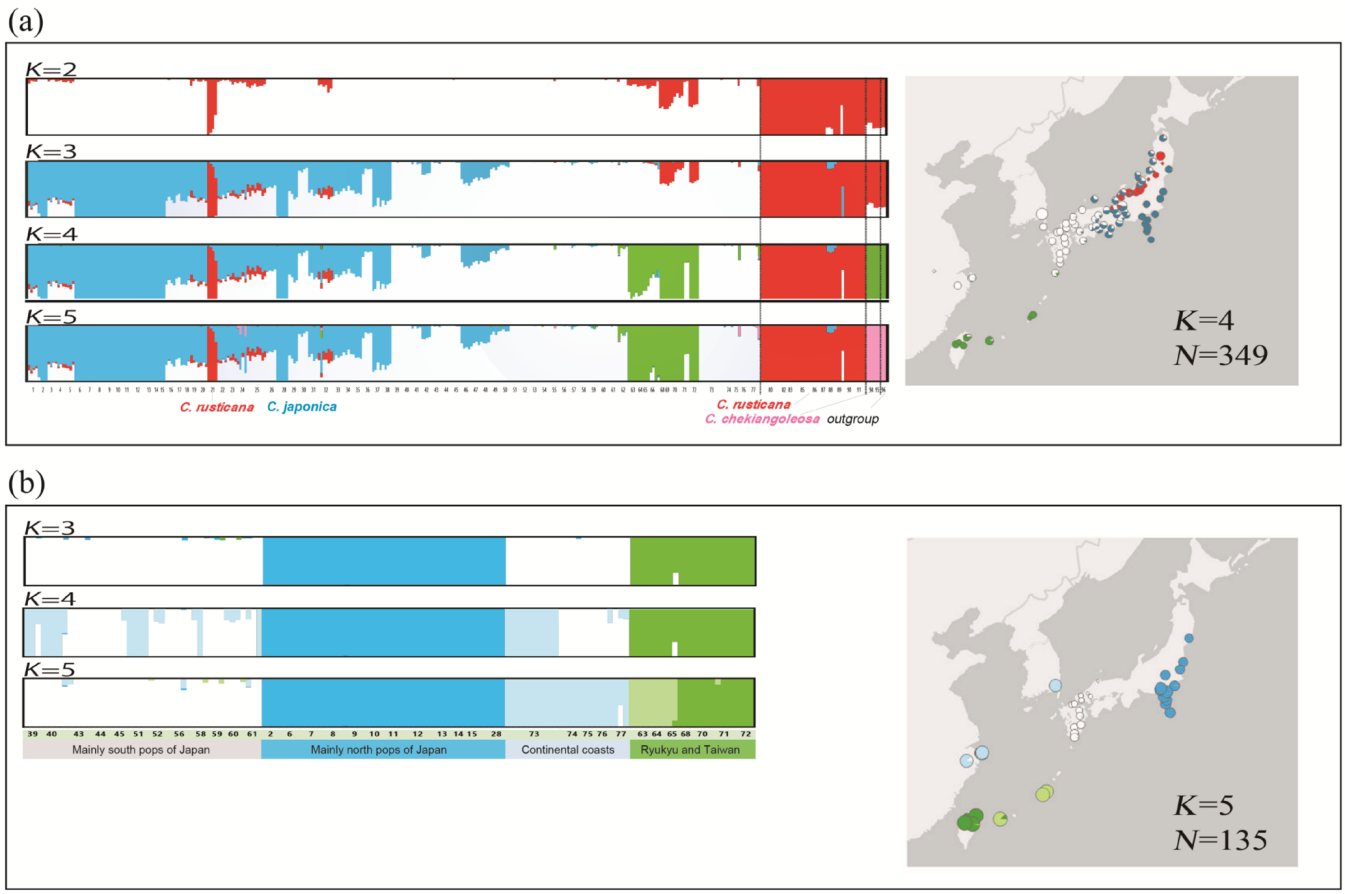
Genetic structure of *Camellia japonica* and *C. rusticana*. (a) Population structure utilising ADMIXTURE (20,462 SNPs). The ancestry proportion for each individual at *K* (the number of clusters) = 2, 3, 4 or 5. The number indicates pop NO. referred from Table S1. Results illustrating ADMIXTURE (*K* = 4) on the map. Optimal *K* values results refer to Figure S5a. (b) Population structure utilising ADMIXTURE of *C. japonica* (13,449 SNPs). The ancestry proportion for each individual at *K* (the number of clusters) = 3, 4 or 5. The number indicates pop NO. Referred from Table S1. Results illustrating ADMIXTURE (*K* = 5) on the map. Optimal *K* values results refer to Figure S5b.

The optimal *K* values were determined to be 2 or 4 (Figure S5a). Although the highest mean posterior probability value was observed for *K* = 4, the results for *K* = 5 remained stable and biologically significant, whereas *K* = 4 did not effectively distinguish the outgroup. Moreover, the outcomes for both *K* = 4 and 5 were nearly indistinguishable (Figure 1a). At *K* = 5, *C. japonica* split into three clusters, while *C. rusticana* and the outgroup formed separate clusters. *Camellia japonica* formed three distinct groups for *K* = 4 and 5: a Northern Japanese, a Southern Japanese (encompassing mainland China and Korea), and a Ryukyu Islands and Taiwan populations (Figure 1a).

**FIGURE 2:**
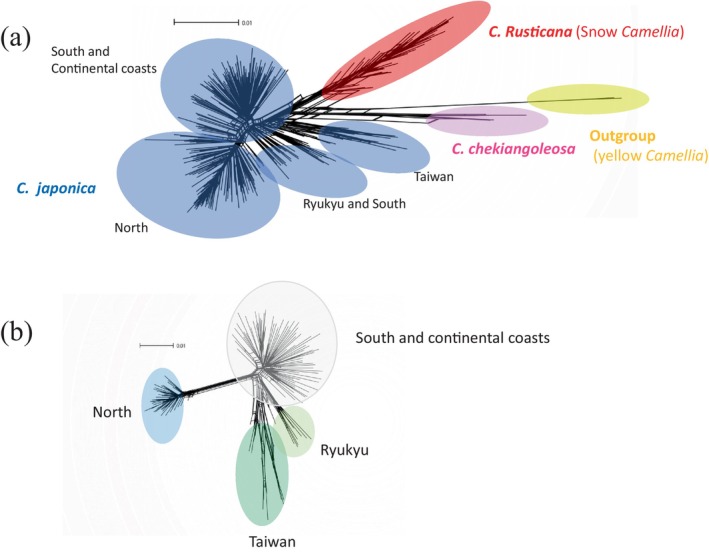
SplitsTree phylogenetic network of *Camellia* species reconstructed using Mig‐seq SNPs data (*R* = 0.5). (a) Indicates three *Camellia* species with outgroup based on 20,462 SNPs and (b) indicates *C. japonica* based on 13,449 SNPs.

In the ADMIXTURE analysis, populations that included individuals with specific clusters below 100% at *K* = 5 were identified as hybrid populations and excluded (Figures 1, 2 and S5b). The samples used for the analysis are listed in column J of Table S1, with blank cells indicating individuals estimated to be hybrids. The five clusters are comprised of the following: the northern Pacific side populations and the southern populations (referred to as ‘North pops’ and ‘South pops’, respectively), the coastline populations of mainland China and Korea (designated ‘Continent pops’), and the Ryukyu Islands and Taiwanese populations (designated ‘Ryukyu‐Taiwan pops’).

### 3.4 Genetic Diversity and Differentiation

The *H*_e_ values for *C. japonica* and *C. rusticana* were approximately identical at 0.077 and 0.072, and the *F*_is_ values were 0.143 and 0.043, respectively. For *C. japonica* populations, The *H_e_* values were 0.103, 0.074, 0.093 and 0.101 in the order of South, North, Continent and Ryukyu‐Taiwan pops. The genetic diversity indicators subjected to population analysis are documented in Table S3. The *F*_st_ value of 0.26 was calculated for each *C. japonica* and *C. rusticana* variable site (Table S4).

Owing to the minute sample size, the Ryukyu Islands and Taiwan pops were analysed as a single population (designated ‘Ryukyu‐Taiwan pops’; *K* = 4). The *H*_e_ values of the North, South, Continent and Ryukyu‐Taiwan pops of *C. japonica* (*R* = 0.5) were 0.074, 0.103, 0.093 and 0.101, respectively (Table S3); the number of private alleles was 906, 1084, 227 and 1000, respectively. The highest and lowest *F*_st_ values were observed between the North and Ryukyu‐Taiwan pops (0.24) and the South and Continent pops (0.04), respectively.

### 3.5 Phylogenetic Networks

The inter‐specific relationships between *C. japonica* and *C. rusticana*, excluding their hybrids (Table S1), were inferred using SplitsTree4 (Figure 2a). Four distinct clusters consistent with the molecular phylogenetic tree (Figure S4) were identified, represented by the outgroups, *C. chekiangoleosa*, *C. rusticana* and *C. japonica* .

Based on the ADMIXTURE analysis with *K* = 4, the results of excluding hybrid individuals between populations are presented in Figure 2b for *C. japonica* . Consequently, they were broadly segregated into North, South, Continent, Ryukyu and Taiwanese populations. Ryukyu Island and Taiwan pops were closely related, with North pops being the farthest apart from the other pops. The result of PCA with ADMIXTURE at *K* = 4 was similar to the results from SplitsTree (Figure 2b; Figure S6). In the PCA plot, the southern and continental pop points were clustered together. Additionally, on the PC1 axis, the North pops were positioned on the positive side, while the other populations were on the negative side, with a substantial contribution of 19.5%. On the PC2 axis, the cluster of South and Continent pops plot along with Ryukyu pops were on the positive side, North pops were on the negative side and Taiwan was broadly plotted on the negative side of the PC2 axis (14.8%). The contribution rate was 60.2%. Pop21 is located at the distribution boundary with *C. rusticana* and has been classified as *C. japonica* based on its distribution and morphological traits. However, Admixture analysis indicated that it belongs to *C. rusticana*, suggesting that it may result from backcrossing after hybridisation (Figure 1).

### 3.6 Estimated Demographic History (DIYABC‐RF)

The ABC method‐based demographic analysis depicted that scenario 2 was selected with the highest probabilities (0.52–0.55; Table S2). The selection probabilities of scenarios 8 and 9 were higher than those of the other scenarios. Additionally, the LDA results portrayed that the scenario 2‐based simulated data corroborated with the observed data. In scenario 2 (MAX generation setting of t 2,000,000), the common ancestors of the three *Camellia* species diverged into *C. chekiangoleosa* and a common ancestor of *C. japonica* and *C. rusticana* (generation number: ts 947,859; 372,984–1,678,620); assuming a generation time of 10 years (Kubayashi et al. 2010), it was estimated to be approximately 10 Ma. Subsequently, at ta 539,516 (135,951–1,122,070), *C. japonica* and *C. rusticana* diverged from their common ancestors (Table S2). The effective population size (*N*) of the common ancestors of the three species (N5) was estimated as 229,660 (Table S2). Since then, *N* has expanded with *C. japonica* , *C. rusticana* and *C. chekiangoleosa* at 855,467, 501,572 and 372,585, respectively (Table S2). Therefore, *C. japonica* , currently the predominantly distributed species, has the largest effective population size.

Demography of the three *C. japonica* species using the most ancestral North pops (excluding inter‐population hybrids) depicted that scenario 2 was supported within a probability range of 0.50–0.55 (Table S2). In scenario 2 (MAX t 2,000,000 setting), the generation (t) was as follows: average ts = 1,182,780 (range 569,304–1,874,390) and average ta = 1,222,500 (range 597,865–1,823,850; Table S2). The effective population sizes (N) for *C. japonica* North pops, *C. rusticana* and *C. chekiangoleosa* were 651,544, 746,903 and 498,873, respectively (Table S2).

For *C. japonica* , an ABC analysis was conducted for North, South, Continent and Ryukyu‐Taiwan pops (Figure 1b) using individuals without inter‐population hybridisation (*K* = 4, Table S1). Owing to the limited sample size, the Ryukyu Islands and Taiwan pops were considered a single population, supported by ADMIXTURE at *K* = 4 (Figure 1b). Among the six scenarios (Figure S3), scenario 4 was selected 285 times and scenario 6 was selected 260 times in 1000 random forest trials, making them the most frequently chosen scenarios. Based on the overall evaluation of compatibility across all scenarios, the posterior probability was highest for scenario 4 (0.693). In scenario 4, divergence occurred between North and South pops, with an average t_3_ of 387,349 (219,467–591,959). The South and Ryukyu‐Taiwan pops diverged with t_2_ 155,283 (60,760–281,858), and the South and Continent pops diverged with t_1_ 44,009 (16,838–75,319) (Table S2). The population sizes for North, South, Continent and Ryukyu‐Taiwan pops were 495,478, 466,012, 616,287 and 469,007, respectively (Table S2).

### 3.7 Estimated Demographic History (fastsimcoal2)

**FIGURE 3:**
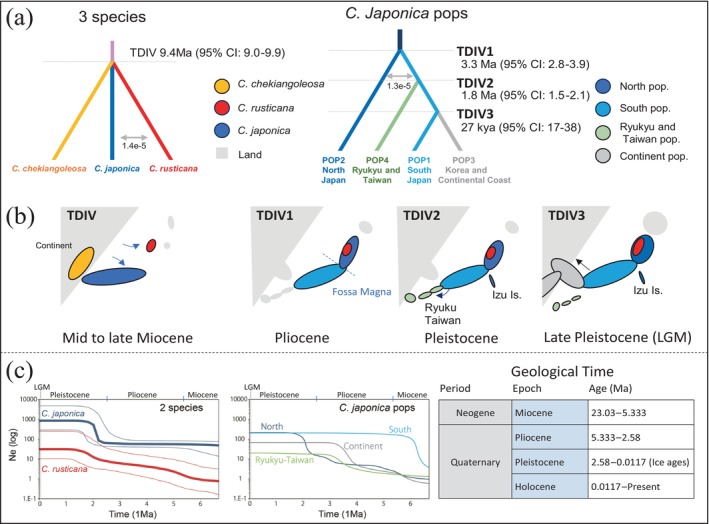
Demographic history of *Camellia japonica* and *C. rusticana*. (a) Best model estimated by fastsimcoal2.7 and (b) the hypothesis of demographic history based on geographic events. Maps are schematic diagrams. The age was calculated using the generation time of *C. japonica* (10 years; Kubayashi et al. 2010). During the Middle Miocene (TDIV), the opening of the Japan Sea separated the Japanese archipelago from the Eurasian continent, introducing *Camellias* to Japan. The TDIV1 period is characterised by the approach and amalgamation of the Fossa Magna (indicated by the blue dashed line), a deep trench in Japan formed by the collision of tectonic plates, facilitating migration from southern to northern Honshu. Subsequently, rapid warming and cooling during the Mid‐Late Pliocene led to the establishment of the genetic structure between the northern and southern populations. TDIV2 corresponds to the Pleistocene glaciation, during which land bridges, including those connecting the Ryukyu Islands and mainland Japan, formed due to a decrease in sea levels. TDIV3 represents the Late Pleistocene, marking the beginning of the last glacial maximum (LGM) and indicating a high likelihood of land bridges forming between Japan and the continent. (c) The demographic population history was reconstructed using the coalescent Bayesian skyline plot (BSP) in BEAST 2.7.7, with the Geological Time shown as a reference in Figure 3.

Based on the results from DIYABC‐RF, we conducted simulations for Scenario 2 (Figure S2) using a model that assumes gene flow between *C. japonica* and *C. rusticana*. Additionally, we also performed simulations using the simpler divergence model of Scenario 10, because Scenario 2 used only the most ancestral North pops of *C. japonica* and showed no differences in divergence times between the three species (Figure S2). As a result, since Scenario 2 did not show convergence in the model, we proceeded with further analyses using Scenario 10. The results for each parameter are shown in Table S5. The divergence time of the three species was approximately mean of 9.6 Ma [95% CI: 7.2–12.7] (Figure 3a,b), which is nearly the same as the divergence time from the common ancestor estimated by the DIYABC model (Table S2), and more recent than the divergence time estimated using the North pops of the *C. japonica* population by the DIYABC model (Table S2). In the simpler divergence model of the two species, the estimated divergence time was 11.5 Ma [95% CI: 11.3–11.6] (Table S5), which is older than the estimate from the three‐species model, though the geological period remains nearly the same. Gene flow between the species was minimal, with 1.6E‐05 from *C. rusticana* to *C. japonica* and 2.5E‐07 in the opposite direction (Table S5).

For *C. japonica* populations, we performed an estimation that included inter‐population gene flow based on Scenario 4 supported by DIYABC‐RF. The divergence time, when considering the generation time of *C. japonica* , between the North and South pops was estimated at approximately 3.3 Ma [95% CI: 2.8–3.9]. The divergence between the South pops and the Ryukyu‐Taiwan pops was estimated at approximately 1.8 Ma [95% CI: 1.5–2.1]. The divergence between the South pops and the Continent pops was approximately 0.027 Ma [95% CI: 0.017–0.037]. Gene flow between populations was 1.28E‐5, which is a low value (Table S5).

### 3.8 Past Population Dynamics Estimated by Expended Bayesian Skyline Plots

When comparing the pops of *C. japonica* from Honshu (North pops and South pops) and *C. rusticana*, *C. japonica* pop began expanding approximately 6 Ma. In contrast, the *C. rusticana* gradually expanded from that time, reaching a peak and stabilising until the present (Figure 3c).

Among the *C. japonica* populations, the South populations experienced rapid expansion of approximately 6 Ma. They maintained a stable population thereafter (Figure 3c). The North populations showed a gradual increase starting at the beginning of the Pleistocene, which began approximately 2.58 Ma, followed by a rapid expansion and subsequent stabilisation until the present (Figure 3c). The Continent and Ryukyu‐Taiwan pops rapidly expanded and stabilised after entering the Late Pliocene, around 4 Ma (Figure 3c). As with the demographic analysis results from DIYABC and fastsimcoal, the estimated effective population sizes were consistent with the sizes predicted based on the current distribution, showing no significant differences in magnitude ( *C. japonica* > *C. rusticana*; South pops > North pops > Continent ≥ Ryukyu‐Taiwan pops in *C. japonica* ; Figure 3c, Table S2 and S5).

### 3.9 Ecological Niche Modelling

**FIGURE 4:**
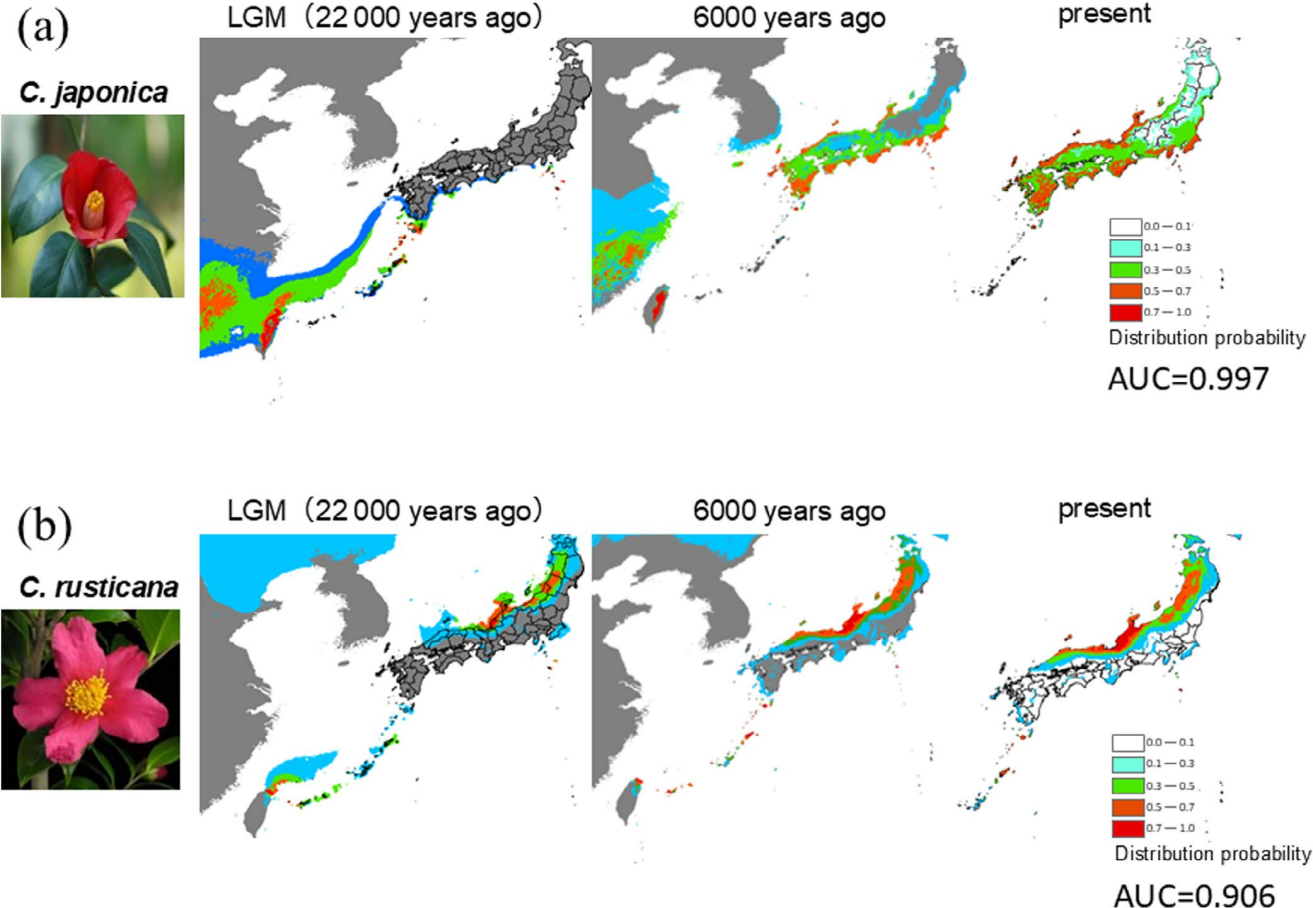
Ecological niche models of (a) *Camellia japonica* and (b) *C. rusticana* using MaxEnt. The environmental variables used were those estimated from the Community Climate System Model 3.0 (CCSM) (Collins et al. 2006) for the last glacial period. A mesh size of 4.5 km was used in WorldClim v. 1.4. The resulting distribution prediction model was evaluated by calculating the area under the curve (AUC) from the ROC analysis. The selection of environmental variables included annual average temperature, average temperature during the four summer months, average temperature during the four winter months, annual precipitation, precipitation during the four rainy months and precipitation during the four dry months. The AUC values calculated by ROC analysis ranged from 0 to 1, with values closer to 1 indicating higher accuracy of the model. The background range and cell size (1 km^2^) of all layers, including the environmental variable values, were uniformly prepared using the ArcGIS software.

Analysis of current habitat suitability using ecological niche modelling revealed that the best‐matched environmental variables were annual average temperature, four‐monthly average temperatures during summer and winter, annual precipitation, and four‐monthly precipitations during the rainy and dry months. The AUC was high at 0.997 for *C. japonica* and 0.906 for *C. rusticana* (Figure 4). The distribution probability for *C. japonica* was remarkably higher (0.7–1.0) in the inland region of Niigata Prefecture, where it is currently non‐existent, while that along the coastal areas of eastern Japan was lower (0.3–0.5) than the actual distribution. The distribution probability of *C. rusticana* in the Chugoku Region (the westernmost region of Honshu), Yamagata and Iwate Prefectures, where it was not originally distributed, escalated to 0.7–1.0. During the LGM (22,000 years before the current distribution), *C. japonica* reportedly extended southward from the Pacific coast of the Izu Islands to Nansei‐shoto (the southwestern islands of Kyushu and the Ryukyu archipelago), with a centre of distribution in southeastern China (Figure 4a). *Camellia rusticana* exhibited reduced distribution along the coasts of Fukui and Ishikawa Prefectures, with high probability (0.7–1.0) along the coast near the border between Niigata and Yamagata Prefectures (Figure 4b).

## 4 Discussion

### 4.1 Validity of Divergence Time Estimates

Demographic history estimation via ABC analysis, assuming a 10‐year generation time, suggests *C. japonica* and *C. rusticana* diverged at approximately 5.4 Ma (95% CI: 1.4–11.2) for all *C. japonica* populations (Max t20000, Table S2) in the best model of Scenario 2. When restricted to the Pacific coast population in northeastern Honshu (North pops), which represents the oldest *C. japonica* origin, the two species diverged approximately 12.2 Ma (95% CI: 6–18.2), indicating a much longer divergence time than previously hypothesised (Table S2). In the fastsimcoal analysis, the divergence time between the three species was estimated to be approximately 9.6 Ma (Figure 3a,b; Table S5), consistent with the results obtained using a simpler model in the ABC analysis, occurring during the Middle to Late Miocene period (Figure 3, Table S5). Additionally, the divergence time estimation among the *C. japonica* populations, using DIYABC, suggested that the initial divergence between the North and South pops occurred around 4 Ma (Table S2). Considering the historical context and the position of the South pops between the North pops and Ryukyu‐Taiwan pops, the model suggesting the initial divergence between the North and South pops is highly plausible. Subsequently, the Ryukyu‐Taiwan pops were estimated to have diverged from the South pops at 1.5 Ma and the continental pops diverged from the South pops at 0.4 Ma. In the fastsimcoal analysis based on this scenario (Figure 3a; Table S5), the divergence between the North and South pops was estimated at 3.3 Ma, between the South and Ryukyu‐Taiwan pops at 1.8 Ma, and between the South and Continent pops at 0.27 Ma. Although these estimates vary slightly from those of the ABC model, they remain within the margin of error. Therefore, the subsequent discussion will be based on the divergence time estimates from the fastsimcoal analysis, which incorporates mutation rates and gene flow between populations.

### 4.2 Process of Ecological Niche Differentiation Between *C. japonica* and *C. rusticana*

#### 4.2.1 Speciation From the Ancestral Species

The Sino‐Japanese floristic region (SJFR), spanning southeastern China to the Korean Peninsula and Japanese mainland, is recognised as a distinct natural floristic area (Takhtajan 1986). Abundant rainfall from the southeastern monsoon characterises this region, fostering rich and diverse plant flora (Harrison et al. 2001; Qiu, Fu, and Comes 2011; Takhtajan 1969, 1986; Wen 1999). The genus *Camellia*, a representative of the SJFR, has estimated origins ranging from 45.4 to 14.3 Ma (Cheng et al. 2022; Rao et al. 2018; Wu et al. 2022; Zhang et al. 2022; Zhao, Hodkinson, and Parnell 2022), with the central estimate occurring in the Middle Miocene.

Numerous biogeographical studies on SJFR plants, including examples of diversification in the middle Miocene, akin to *Camellia*, are evident in various genera, such as *Cardiocrinum* (Liliaceae) (Lu et al. 2020), *Euptelea* (Eupteleaceae) (Cao et al. 2016) and *Diabelia* (Caprifoliaceae) (Wang et al. 2020). For *Cardiocrinum*, molecular phylogenetic analysis calibrated with fossils shows *C. giganteum* diverging from the common ancestor of *C. cathayanum* and *C. cordatum* at approximately 9.08 Ma, with subsequent divergence between these two species at approximately 5.09 Ma (Lu et al. 2020). This divergence aligns with the Middle Miocene, resembling the differentiation period between continental *Camellia* (*C. chekiangoleosa*) and the Japanese *Camellia* clades investigated in this study. These findings suggest a comparable trend in the timing of divergence in the Japanese *Camellia*.

Considering the influence of Middle Miocene geological and climatic events on temperate plants, including the genus *Camellia*, in the SJFR, the uplift of the Qinghai‐Tibet Plateau, major river basin reorganisations in southwestern China, the complete separation of the Japanese archipelago from Eurasia and ongoing aridification since the late Middle Miocene have assumed pivotal roles in the diversification of temperate plants in the SJFR (Cao et al. 2016; Meng et al. 2017; Zheng et al. 2004). In the Early‐to‐Middle Miocene, East Asia witnessed elevated temperatures and precipitation, fostering a humid, warm climate and strengthening the summer monsoon system (Sun and Wang 2005). The genus *Camellia*, comprising evergreen broadleaf trees in the temperate zone, is widely distributed in the present‐day SJFR and was dispersed throughout the region during this period (Yang et al. 2017). These geological and climatic events have also influenced speciation and diversification. The estimated divergence time between *C. chekiangoleosa*, *C. japonica* and *C. rusticana* occurred at approximately 9.6 Ma (Figure 3a; Table S5) during the Middle Miocene. This suggests that as the Japanese archipelago split from the Eurasian continent and the Sea of Japan expanded, *C. japonica* and *C. rusticana* diverged from their continental relative, each adapting to the southern and northern fragments, respectively. The divergence of the common ancestors of *C. japonica* and *C. rusticana* may be attributable to geological isolation associated with the separation of the continent and the Japanese archipelago rather than being primarily driven by climatic events (Figure 3b).

#### 4.2.2 Ecological Niche Differentiation

Subsequently, focusing on climatic influences, the global cooling trend during this period likely facilitated the diversification of the sect. *Camellia* on the continent (Rao et al. 2018). Considering the CI in divergence time estimates, the Japanese species possibly diverged during this period on the continent. Additionally, BSP analysis indicated that the effective population size of *C. rusticana* slowly expanded following its divergence, with the population gradually increasing in size as the Japanese archipelago expanded. Considering that *C. rusticana* exhibits distinct morphological and ecological traits adapted to snowy environments compared to *C. japonica* (Abe, Miura, and Motonaga 2021), these attributes are likely adaptations to the harsh snowy conditions. The ENM (Figure 4b) shows that the distribution of *C. rusticana* has remained relatively stable from the last glacial period to the present, consistent with the BSP results, suggesting that *C. rusticana* has consistently inhabited the snowy regions along the Sea of Japan. The formation of these harsh snowy environments is estimated to have occurred approximately 1.7 Ma in the Quaternary (Igarashi et al. 2018), and *C. rusticana* adapted to this environment following its divergence.

The elements of Japan Sea provided an opportunity for exploring the driving mechanism of ecological niches to morphological divergence and speciation. Plant species unique to the Sea of Japan region, known as Japan Sea elements, are distributed according to snowfall levels (Maekawa 1977). The observation that species closely related to Japan Sea elements are found in the warm temperate zones of the Pacific region suggests their adaptation to past climate changes under Quaternary glaciation, specifically in temperate environments in Japan (Hotta 1974; Sakai 1982). A total of 212 taxa of Japan Sea elements and their relatives, including *C. japonica* and *C. rusticana*, have been documented (Honda 1950; Sato 2005). *C. rusticana* presumably adapts to snowy regions and specialises in *C. japonica* (Tsuyama 1956; Tsuyama, 1998). However, contrary to the conventional hypothesis that *C. rusticana* evolved from *C. japonica* as an adaptation to Quaternary glaciation, our results showed that the two species diverged much earlier. Previous studies have shown that both species exhibit significant independence in terms of leaf morphology, filament characteristics and pigment composition (Abe, Miura, and Motonaga 2021; Mori, Hasegawa, and Moriguchi 2023). In our study, *H*_*e*_ values did not differ significantly between the species (Table S3), indicating that *C. rusticana* is not influenced by genetic drift or bottleneck associated with speciation from *C. japonica* , thus supporting their separation. As a result, our findings support Honda's (1950) conclusion that *C. rusticana* and *C. japonica* are distinct species.

### 4.3 *C. japonica* Demography and Historical Background

Summarising our *C. japonica* research, the molecular phylogenetic tree (RA × ML) highlights significant differentiation in Taiwan pops and Ryukyu pops, in contrast to the geographically contiguous clustering of Honshu pops (Figure S4). SplitsTree and PCA, excluding the hybrids, further confirmed the distinct separation of North pops from the others (Figures 2 and S5). The difference from RA × ML analysis is likely due to the exclusion of hybrid individuals, which resulted in a more clearly defined genetic structure. Based on the DIYABC and fastsimcoal demographic estimations, which analysed populations divided into North, South, Continent and Ryukyu‐Taiwan groups, it was estimated that the North pops diverged from the South pops around 3.3 Ma and have been maintained as an ancient population (Figures 3a,b and S5). This suggests that *C. Japonica* populations in the Japanese archipelago did not consist of groups that migrated south during the glacial period or north during the interglacial periods. Alternatively, this suggests the presence of a large ancestral population that persists in the northern regions. BSP analysis of past effective population size changes indicated that the South pops expanded after 6 Ma, and the Continent pops and Ryukyu‐Taiwan pops expanded in the Late Pliocene (after 4 Ma). The North pop expanded after the Pleistocene and then stabilised (Figure 3). The current effective population sizes are considered appropriate when estimated based on the current distribution areas. Considering the results of the fastsimcoal on distribution changes, we explored historical contexts and distinguishing periods: (1) ancestral North Pop divergence; (2) Ryukyu‐Taiwan pops divergence; and (3) South and Continent pop divergence (Figure 3b), compared with molecular phylogenetic studies from the late Middle Miocene onwards.

#### 4.3.1 Period of Ancestral North Pop Divergence

Using fastsimcoal analysis, the initial divergence within *C. japonica* populations, separating the North and South pops, occurred at approximately 3.3 Ma during the Pliocene (Figure 3a,b; Table S5). Around 3.3 million years ago, a significant warming event known as the Mid‐Pliocene warmth occurred, with temperatures rising by 2°C–3°C, followed by substantial cooling associated with the expansion of the Northern Hemisphere ice sheets (Sarnthein et al. 2009). Bartoli et al. (2005) refer to these late Pliocene environmental changes as the ‘Climate Crash’, which may have influenced the genetic structure formation between the North and South pops of *C. japonica* . BSP analysis indicates that after the effective population size of *C. japonica* expanded around 2 Ma at the beginning of the Pleistocene (Figure 3c). This stability is likely due to the inclusion of many Izu Islands individuals within the North pops used in the analysis, which probably expanded their distribution based on the formation of the Izu Islands post‐Pleistocene. Furthermore, the Izu Islands populations may have functioned as refugia during the Pleistocene glaciations, allowing them to maintain a stable population size unaffected by the glacial cycles.

Many cases show distinct genetic structure differences between northern and southern populations of closely related species in the Japanese archipelago (e.g., Kirihara et al. 2013). This phenomenon has been observed in populations of the same species, such as *C. japonica* . Research in this field has often examined analogous cases within animal populations, such as *Luciola cruciata* (Suzuki, Sato, and Ohba 2002) and the mayfly *Isonychia japonica* (Tojo et al. 2017), in which divergence between the northern and southern regions hypothetically occurred millions of years ago. Recent estimates suggest species divergence since the Middle Quaternary, which is supported by various studies (Iwasaki et al. 2012; Sakaguchi et al. 2011; Yoichi et al. 2023), with higher *H*_*e*_ in southwestern Japan and lower diversity in northern populations (Hiraoka and Tomaru 2009; Takahashi et al. 2002; Tomaru et al. 1997, 1998; Iwasaki et al. 2012; Sakaguchi et al. 2011; Tamaki et al. 2019; Yoichi et al. 2023). Larger southern populations tended to exhibit higher *H*_*e*_, whereas northern migration or refugia populations tended to have lower *H*_*e*_ (Tsumura 2022). Similar to *Camellia*, *Magnolia kobus* provides an illustrative example (Tamaki et al. 2019). Similar to *C. japonica* , *Magnolia kobus* diverged into northern and southern lineages, with significantly lower *H*_*e*_ in the northern lineage. This pattern is commonly observed in tree species in Europe and North America (Comps et al. 2001; Konnert and Bergmann 1995; Liepelt et al. 2009; Magri et al. 2006; McLachlan, Clark, and Manos 2005). However, the estimated divergence time of these lineages was approximately 565,000 years, suggesting the impact of Quaternary glacial cycles on the genetic differentiation between the northern and southern regions. In our study, the ancestral population of *C. japonica* persisted in the northern region, showing ancient divergence before the Quaternary period. This implies that the genetic structures of the northern and southern populations are older and were established prior to the Pleistocene, differing from what has been debated in previous studies.

#### 4.3.2 Period of Ryukyu‐Taiwan Pop Divergence

Subsequently, the separation of the Ryukyu‐Taiwan pops from the southern pops occurred at approximately 1.8 Ma (Figure 3a). The Ryukyu archipelago has experienced repeated connections and separations from the Asian continent, Taiwan and Kyushu since the Neogene period owing to crustal movements and changes in sea levels (Nakamura 2012). However, by the early Pleistocene (approximately 1.7 Ma), the archipelago was segregated into Northern Ryukyu, Central Ryukyu and Southern Ryukyu because of the formation of two deep‐sea straits (Tokara Strait and Kerama Gap) created by subsidence of the seafloor, each reaching a depth of over 1000 m. This timeframe aligns with the differentiation period indicated by the findings of this study (Figure 3b).

We also explored the link between the continent and the Japanese archipelago via Taiwan and the Ryukyu Islands. Since the Late Middle Miocene, this connection can be categorised into three stages, with large portions of the East China Sea (ECS) seabed exposed around 7.0–5.0 Ma, 2.0–1.3 Ma and 0.2–0.015 Ma. Land bridges formed during the Quaternary between Taiwan and the Ryukyu Islands as well as between the Chinese and Japanese mainlands during glacial periods (Kimura 1996; Kizaki and Oshiro 1977, 1980; Ujiie 1990). Although many phylogeographic studies have proposed a close relationship between species or within a species between Taiwan and the Chinese mainland, indicating historical gene flow through a connected landmass in the Quaternary (Chiang and Schaal 2006; Chiang et al. 2006; Huang, Hwang, and Lin 2002; Mitsui et al. 2008; Qi et al. 2014), our study revealed that *C. japonica* populations in the Ryukyu‐Taiwan population diverged from the Japanese mainland population (Figure 3b), suggesting no introduction from the Chinese mainland to these regions (*F*st values of 0.16, between the Ryukyu‐Taiwan pops and the mainland, and 0.04 between the South pops and the mainland). Despite the formation of a land bridge in the Quaternary between mainland China and Taiwan, movement between these regions seems unlikely. A possible explanation for this is the presence of environmental barriers between mainland China and the Taiwan‐Ryukyu Islands. Grasslands or semi‐arid temperate forests likely covered the interconnected land bridge regions in the Quaternary, whereas *C. japonica* thrives in humid forests, possibly acting as a barrier between the mainland and these areas (Harrison et al. 2001; Ray and Adams 2001). Furthermore, this study hints at the differentiation between Taiwan and the Ryukyu Islands, influenced by the division of the Ryukyu Archipelago into North, Central and South Ryukyu from the late Neogene to the early Quaternary. However, the divergence time between the Ryukyu and Taiwanese populations was not estimated in this study, because of the limited number of samples from each island. Hence, we recommend investigating it in the future with a more comprehensive sample collection. Further detailed analyses may elucidate the relationship between the land bridge connecting the mainland and Kyushu and its role in isolation and evolution. The effective population size estimated by BSP expanded by approximately 4 Ma and has remained stable since. The fastsimcoal analysis indicates a divergence from the southern population of approximately 1.8 Ma. This suggests that initial migration occurred from the southern population during the early connection period, with no subsequent migrations during later connection periods, leading to further genetic differentiation as a population. However, because this population did not experience the bottleneck effects of island isolation and population size reduction and also maintained large genetic diversity (Table S3), as seen in other species, it can be attributed to the establishment of a stable niche earlier than other species due to the founder effect (e.g., Frankham 1997), allowing it to maintain its position.

#### 4.3.3 Period of South and Continent Pop Divergence

The distinction between the South Japanese and Continental populations (Figure 3a,b) occurred at approximately 27 Kya during the Quaternary glacial period. Between the Marine Isotope Stage 12 and 10 interglacial periods, a land bridge formed between the Japanese archipelago and the Korean Peninsula, which coincided with the periodic opening of the Tsushima/Korean Strait between South Japan and Korea within a 3.5–1.7‐million‐year interval (Kitamura and Kimoto 2006). Numerous reverse‐colonisation occurred from the Japanese archipelago to the Asian continent in response to LGM climatic changes during land bridge formation (e.g., Qiu, Fu, and Comes 2011). For instance, molecular clock‐based and geological evidence and cpDNA data for the genus *Kirengeshoma* suggest a relatively ancient vicariant divergence of *K. koreana* in the Japan–Korea region at the Plio‐Pleistocene boundary (approximately 2.25 Ma). In contrast, the latter migrated into China during the early‐to‐mid Pleistocene via the ECS basin (Qiu et al. 2009). Similar phenomena were observed in genome‐wide analyses of the aquatic insect *Appasus japonicus* , revealing lineages that diversified in the Japanese archipelago and underwent back dispersal to the continent (Suzuki et al. 2021; Tojo et al. 2017). During this period, the *Camellia* population also migrated southward from the Japanese archipelago to the continent (Figure 3b). ENMs analysis indicated a southward shift in optimal habitats for *C. japonica* , signifying movement to the continent during the land bridge emergence (Figure 3b). The estimated continental population size, ranging from Korea to the coastal side of China, was more significant than that of the other Japanese populations (Table S2). However, the continental population exhibited fewer private alleles and relatively low *H_e_* (Table S3), suggesting a greater likelihood of a migrant population. These results depict that the Chinese and Korean populations originated from southern Japan (Figure 3b).

### 4.4 The Genetic Relationship Between Japanese and Continental *Camellias*

First, we summarise previous studies on the molecular phylogenetic relationships between the Japanese *Camellias*, the continental sect. *Camellia*, the closest section to sect. *Camellia*. Previous research sheds light on the evolutionary dynamics within these taxa (e.g., Vijayan, Zhang, and Tsou 2009; Pang et al. 2022; Zhao, Hodkinson, and Parnell 2022; Cheng et al. 2022; Qin et al. 2023; Zan et al. 2023; Yan et al. 2021). For example, Vijayan, Zhang, and Tsou (2009) analysed nrITS sequences of 112 species, revealing that *C. chekiangoleosa* is ancestral, giving rise to two clades: one including *C. rusticana* (along with *C. azalea* and *C. edithae* ) and the other including *C. japonica* (with *C. fluviatilis* and *C. brevistyla* ). This synthesis supports the notion that a common ancestor between the clades of *C. rusticana* and *C. japonica* derived from *C. chekiangoleosa* exists. However, Pang et al. (2022) suggest a more ancestral position for *C. rusticana* compared to *C. oleifera* belonging to the sect, found in their Bayesian inference phylogenetic tree from chloroplast DNA regions. Zhao, Hodkinson, and Parnell (2022) positions *C. chekiangoleosa* and *C. japonica* as sister species. Cheng et al. (2022) demonstrated that *C. azalea*, belonged to the same sect. *Camellia* as *C. chekiangoleosa*, as a sister group, with *C. japonica* branching off 15 Ma. Qin et al. (2023) suggest the integration of the sect. *Oleifera* into the sect. *Paracamellia* based on nuclear gene analysis. From this study, differences are observed between the molecular phylogenetic trees based on plastomes and the 982 SCH genes. The age estimation of terminal species is much younger in the plastome tree than in the SCH gene tree. However, *C. japonica* and *C. chekiangoleosa* are closely related, with *C. chekiangoleosa* occupying an ancestral position despite having short branch lengths. Additionally, the sect. *Paracamellia* branched off later than *C. japonica* (Qin et al. 2023). Zan et al. (2023) compare the concatenated tree and the coalescent tree of 87 *Camellia* species, aligning with Qin et al. (2023) in positioning *C. japonica* as more ancestral than *C. oleifera* and *C. sasanqua* (sect. *Oleifera*). Similarly, Yan et al. (2021) suggest that *C. japonica* diverged from *C. chekiangoleosa* approximately 10 Ma, consistent with the findings of this synthesis. Additionally, *C. japonica* is identified as the closest sister species to *C. lutchuensis*, the seat. *Theopsis* species is found exclusively in the Ryukyu Islands, with *C. chekiangoleosa* branching off shortly thereafter (though with short branch lengths). In the final section, we will discuss the issue of species from different sections being grouped in the same clade. In the study by Wu et al. (2022) on the chloroplast genome sequences of 26 *Camellia* plants, *C. chekiangoleosa* diverges from *C. sasanqua* within the sect. *Oleifera*. Subsequently, *C. japonica* and *C. oleifera* separate as sister species from *C. chekiangoleosa*. Similarly, Rao et al. (2018) shows a clade, including *C. japonica* (along with *C. fluviatilis* and *C. brevistyla* ), derived from *C. chekiangoleosa*, indicating a more ancestral position for *C. chekiangoleosa*. In summary, despite variations in the inferred phylogenetic relationships of the *Camellia* genus in previous studies, *C. chekiangoleosa* is likely to be ancestrally positioned among the three species and its close relationship with *C. japonica* is indisputable.

Regarding the estimation of divergence times, the chronological order is summarised based on previous studies and the divergence dates between the sect. *Camellia* and its closely related sections, the sect. *Oleifera* and the sect. *Paracamellia* are as follows. Zan et al. (2023) reported that the divergence between *C. japonica* and *C. sasanqua* occurred approximately 6 Ma, with the two species forming a sister group. In Wu et al. (2022), the divergence between the sect. *Camellia* and *C. oleifera* are estimated to be 5.88 Ma. Zhao, Hodkinson, and Parnell (2022) propose a divergence of 19 Ma between the sect. *Camellia* and the sect *Paracamellia*. In their study, *C. japonica* and *C. chekiangoleosa* are identified as sister species with a divergence estimated at 9.9 Ma, which aligns closely with the estimates in the current research. Additionally, Zan et al. (2023) suggest a divergence of approximately 16–17 Ma between *C. japonica* and *C. chekiangoleosa*. Other studies provide varying estimates, such as Cheng et al. (2022), indicating a divergence of *C. japonica* from the clade containing *C. chekiangoleosa* around 15 Ma, Rao et al. (2018) suggesting a divergence of *C. chekiangoleosa* around 10 Ma Yan et al. (2021) proposing a slightly earlier divergence, and Qin et al. (2023) suggesting the emergence of a clade containing *C. japonica* around the Last Glacial Maximum of the fourth glaciation period. In our study, the fastsimcoal model estimates the divergence at 9.6 Ma among three species and 11.5 Ma between *C. japonica* and *C. rusticana*.

Summarising the above, *C. chekiangoleosa* is likely to be ancestral; however, the position of *C. rusticana* remains unclear. Though limited in number, examining previous studies that include *C. rusticana* could provide insights into this matter. Tanikawa et al. (2008) investigated the PCR‐RFLP band patterns, finding standard bands between *C. chekiangoleosa* and *C. rusticana*, yet none of them were found with *C. japonica* . As mentioned above, Vijayan, Zhang, and Tsou (2009) suggested *C. rusticana* as a sister group to *C. azalea* and *C. edithae* , indicating a closer relationship to *C. chekiangoleosa* than *C. japonica* . Pang et al. (2022) placed *C. rusticana* and *C. oleifera* ancestrally within the sect. *Camellia* clade, while *C. japonica* varieties and *C. chekiangoleosa* were derived. Rao et al. (2018) proposed a clade consisting of *C. azalea* and *C. edithae, which* has diverged from *C. chekiangoleosa*. Cheng et al. (2022) found that *C. chekiangoleosa* and *C. azalea* form a sister group. In summary, if *C. rusticana* is closely related to *C. chekiangoleosa*, *C. azalea* and *C. edithae* , *C. rusticana* is suggested to be ancestrally positioned compared to *C. japonica* . However, the branch lengths were short, indicating uncertainties in the relationships among these species. Taking these previous studies and our results into consideration, both *C. japonica* and *C. rusticana* may have diverged from *C. chekiangoleosa* without a significant time gap between the two species.

### 4.5 Questions and Future Directions From Previous Studies

Summarising the aforementioned previous studies, numerous instances have occurred where geographically proximate species cluster together in the same clade despite belonging to different sections. This tendency for geographically proximate species to cluster in the same clade suggests the influence of natural hybridisation, specifically given the overlapping distribution of *C. japonica* with these species. Additionally, considering the common utilisation of members of the genus *Camellia* as horticultural varieties and the use of wild species as progenitors for cultivated varieties, the impact of artificial hybridisation is also plausible. The samples utilised in previous studies include specimens from the wild and also those from botanical gardens, and many analyses lack the verification of phylogenetic relationships with geographical information. Therefore, in future studies, organising the molecular phylogeography in conjunction with morphological and geographical data is necessary. Additionally, considering the history of hybridisation, reevaluating molecular phylogenetic relationships is warranted.

## Author Contributions

**Harue Abe:** conceptualization (lead), data curation (equal), formal analysis (lead), funding acquisition (equal), investigation (lead), methodology (lead), project administration (equal), resources (equal), software (equal), supervision (lead), validation (lead), visualization (lead), writing – original draft (lead), writing – review and editing (lead). **Saneyoshi Ueno:** conceptualization (equal), data curation (equal), formal analysis (supporting), funding acquisition (equal), investigation (supporting), methodology (supporting), project administration (supporting), resources (equal), software (supporting), supervision (supporting), validation (supporting), visualization (supporting), writing – original draft (supporting), writing – review and editing (supporting). **Ayumi Matsuo:** data curation (supporting), formal analysis (supporting), software (equal), writing – original draft (supporting), writing – review and editing (supporting). **Shun K. Hirota:** formal analysis (supporting), software (equal), writing – original draft (supporting), writing – review and editing (supporting). **Hiroki Miura:** conceptualization (supporting), data curation (supporting), formal analysis (supporting), investigation (supporting), resources (supporting), software (supporting), validation (supporting), visualization (supporting), writing – original draft (supporting), writing – review and editing (supporting). **Mong‐Huai Su:** data curation (supporting), investigation (supporting), resources (supporting), writing – original draft (supporting), writing – review and editing (supporting). **Yun‐Guang Shen:** data curation (supporting), investigation (supporting), resources (supporting), writing – original draft (supporting), writing – review and editing (supporting). **Yoshihiko Tsumura:** data curation (supporting), funding acquisition (equal), resources (supporting), writing – original draft (supporting), writing – review and editing (supporting). **Yoshihisa Suyama:** resources (supporting), software (supporting), supervision (supporting), writing – original draft (supporting), writing – review and editing (supporting). **Zhong‐Lang Wang:** data curation (supporting), resources (supporting), supervision (supporting), writing – original draft (supporting), writing – review and editing (supporting).

## Conflicts of Interest

The authors declare no conflicts of interest.

## Supporting information


Figures S1–S6

Tables S1–S5

## Data Availability

The MIG‐seq data are available in the DNA Data Bank of Japan (DDBJ) Sequence Read Archive: DRA017786.
