# Supplementary material for: Evolutionary Histories of Camellia japonica and Camellia rusticana
Source: Ecol Evol. 2024 Dec 24;14(12):e70721. doi: 10.1002/ece3.70721 (PMC11667151; doi:10.1002/ece3.70721)
Supplement: Supplementary file 1 — Figures S1–S6 [file ECE3-14-e70721-s002.pdf]

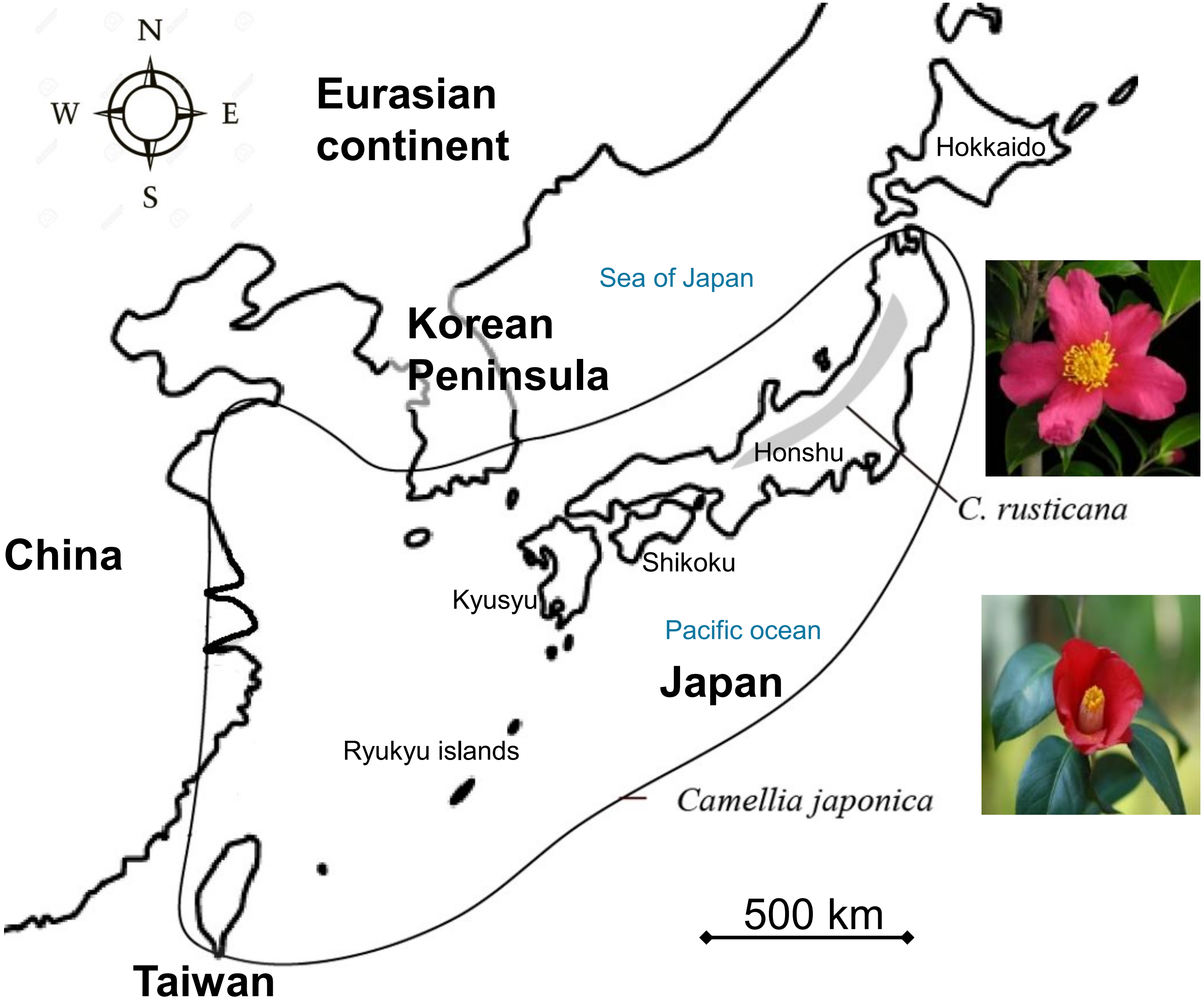

Fig. S1 Distribution of the sect. *Camellia* in Japan.

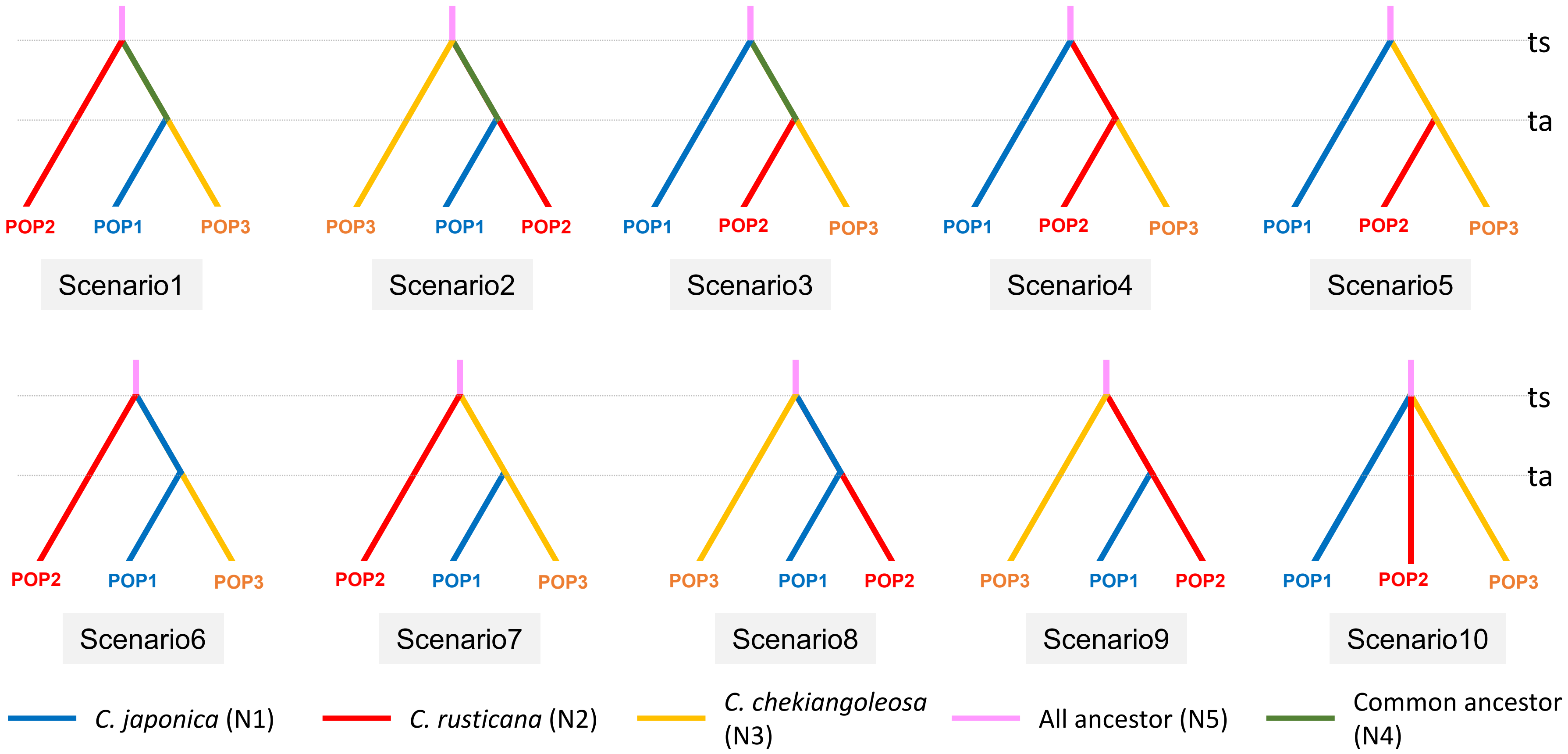

Fig. S2 Design of DIYABC-RF analysis to compare the ten demographic scenarios of *Camella japonica* and *C. rusticana*.

POP1: *C. japonica* (blue bars), POP2: *C. rusticana* (red bars), and POP3: *C. chekiangoleosa* (yellow bars). Hybrid individuals were excluded through ADMIXTURE analysis, and the scenarios assumed no hybridization. Demographic and historical parameters, including five effective population sizes (N1, N2, N3, N4, and N5) indicated by bars of different colors, along with two divergence time events (ts and ta) (refer to Table S2).

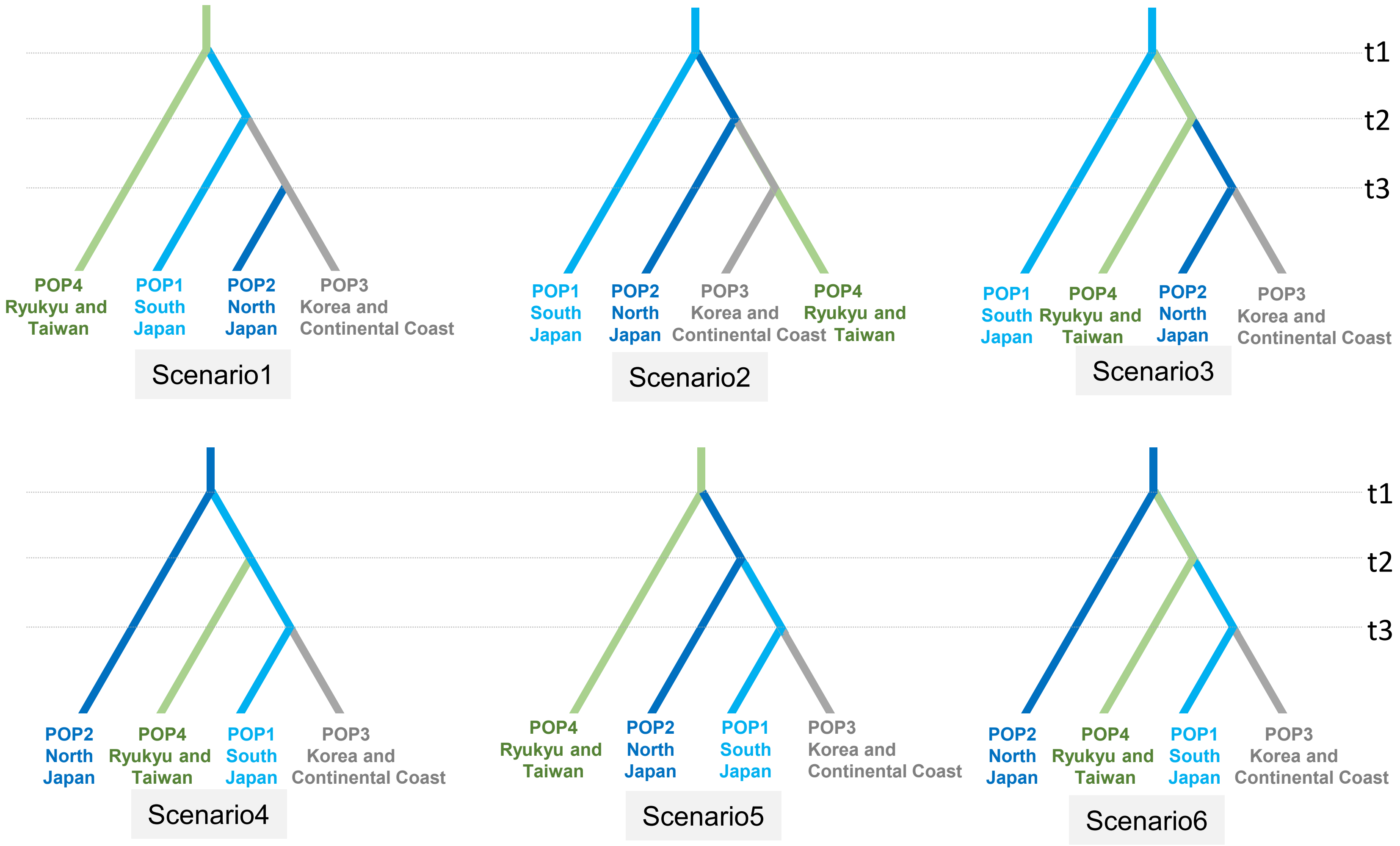

Fig. S3 Design of DIYABC-RF analysis to compare the six demographic scenarios of *Camella japonica.*

POP1: South pops., POP2: North pops., POP3: Continent pops., and POP4: Ryukyu-Taiwan pops.. Hybrid individuals were excluded through ADMIXTURE analysis, and and the scenarios assumed no hybridization. Demographic and historical parameters, including five effective population sizes (In order from POP1 to POP4, they are N1, N2, N3, and N4) indicated by bars of different colors, along with three divergence time events (t_1, t_2 and t_3) (refer to Table 2).

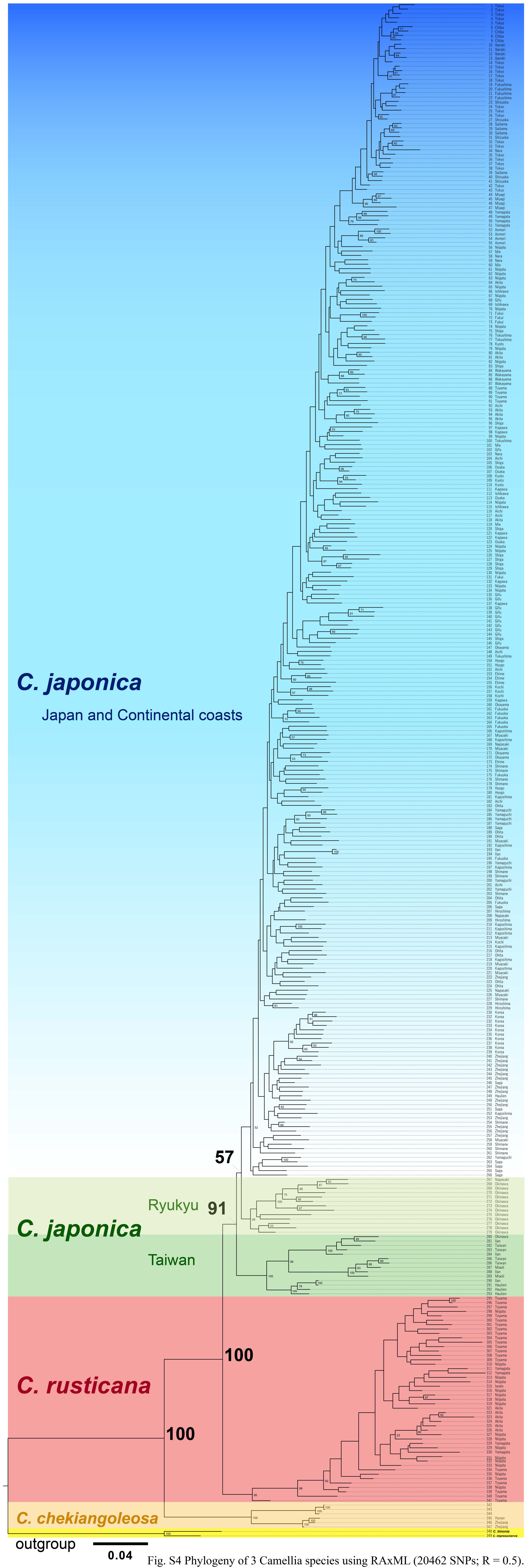

Fig. S4 Phylogeny of 3 Camellia species using RAxML (20462 SNPs; R = 0.5).

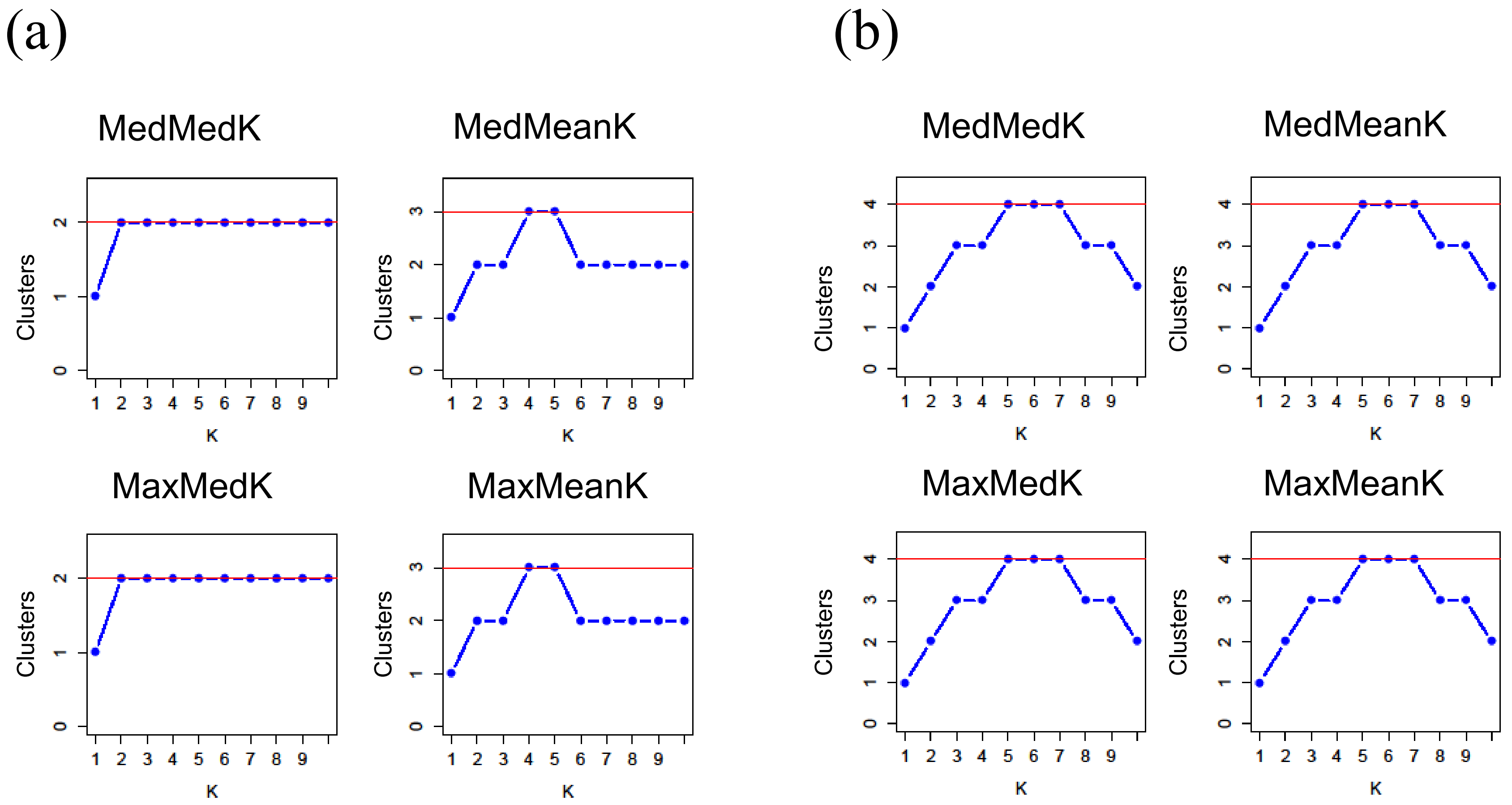

Fig. S5 Optimal K values results of ADMIXTURE analysis.

(a) *Camellia japonica* and *C. rusticana* (refer to Fig. 1a). (b) *Camellia japonica* (refer to Fig. 1b). MedMedK, MedMeanK, MaxMedK, and MaxMeanK were estimated post-simulation using the online software Structure Selector (Li & Liu, 2018) following the Puechmaille method (Puechmaille, 2016).

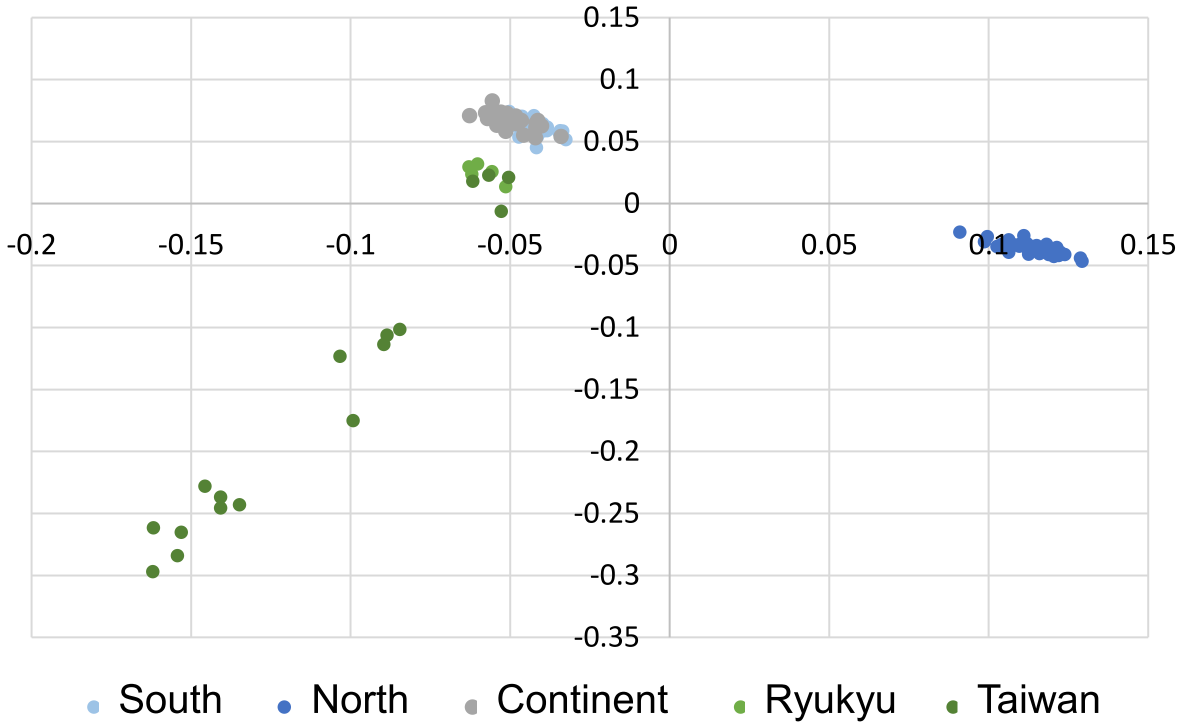

Fig. S6 Principal component analysis (PCA) of five populations from *Camellia japonica* based on Mig-seq SNPs data (13449 SNPs; R = 0.5).
